# Rare Case of Adult Undifferentiated (Embryonal) Sarcoma of the Liver Treated with Liver Transplantation: Excellent Long-Term Survival

**DOI:** 10.1155/2012/519741

**Published:** 2012-09-27

**Authors:** Renumathy Dhanasekaran, Alan Hemming, Elaine Salazar, Roniel Cabrera

**Affiliations:** ^1^Department of Medicine, University of Florida, Gainesville, FL, USA; ^2^Department of Surgery, University of California, San Diego, CA 92103, USA; ^3^Department of Pathology, University of Florida, Gainesville, FL 32610, USA; ^4^Division of Gastroenterology, Hepatology and Nutrition, University of Florida, Gainesville, FL 32610, USA

## Abstract

We present the case of a 54-year-old gentleman who presented with abdominal distension and a CT scan of his abdomen revealed a large (25 cm) left hepatic lobe tumor. He received chemotherapy for over 1.5 years. The CT scans at the completion of this therapy revealed that the tumor had actually slightly grown in size. He underwent orthotopic liver transplantation without any major complications. The explant histopathology revealed an undifferentiated embryonal cell sarcoma (UECS) composed of relatively bland spindled cells arranged in short fascicles. It is now 10 years and 3 months since his last transplant and the patient remains well with no tumor recurrence.

## 1. Case Report

This patient is a 54-year-old gentleman who presented with abdominal distension and a CT scan of his abdomen revealed a large tumor (25 cm) in the left hepatic lobe. Laboratory data showed normal liver function tests, negative viral hepatitis markers, and normal tumor markers for AFP, CEA, and CA 19-9. On imaging, the liver tumor involved all three hepatic veins, pressed on the porta hepatis, splayed out the right portal vein, and compressed the right portal vein. He had evidence of portal hypertension with an enlarged spleen and varices in the splenic hilum, collateral vessels within the abdominal walls, and a recanalized umbilical vein. CT scan of the chest and bone scan did not reveal metastatic lesions. A percutaneous biopsy was read as probable primary angiosarcoma of the liver in February 1999 at the presenting hospital. A resection was not performed due to extent of vascular involvement and presence of portal hypertension. An extended course of chemotherapy was attempted initially with adriamycin and ifosfamide for ten months, then one session of chemoembolization followed by dacarbazine for another eight months. Imaging at the completion of this therapy revealed that the tumor had slightly grown in size, but no metastatic lesions were identified. Since he had failed extended chemotherapy and the tumor remained unresectable, he was offered liver transplantation. He underwent orthotopic liver transplantation in March of 2002 without any major complications. 

**Figure 1:**
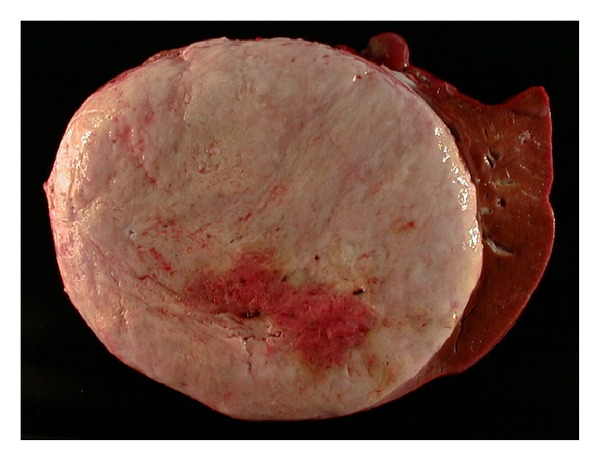
Gross appearance of tumor in the explant, tumor mass occupied the majority of the left hepatic lobe.

**Figure 2:**
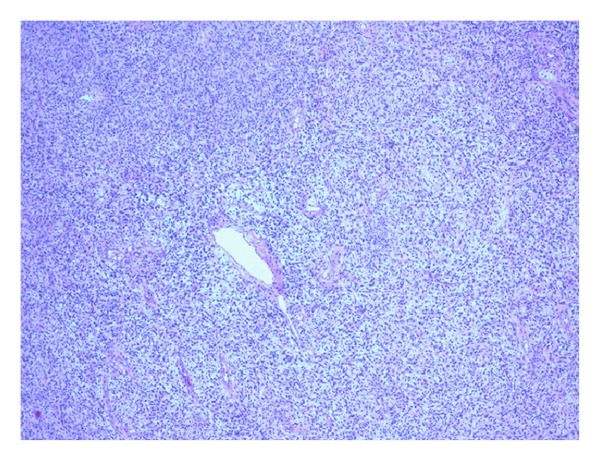
Low-power field histopathology shows undifferentiated spindle cell sarcoma with bland spindled cells arranged in short fascicles.

**Figure 3:**
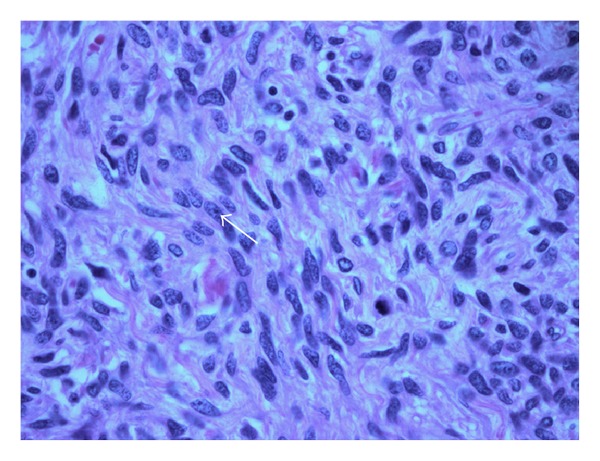
High-power field histopathology shows well-preserved tumor with mitotic figures (arrow).

The explant liver weighed 8.05 kilograms and measured 37.5 cm (medial to lateral), 32.0 cm (superior to inferior), and 17.0 cm (anterior to posterior). Serial sectioning of the liver revealed a 30.0 × 22.0 × 21.0 cm well-circumscribed, white-tan to light brown mass with a central area of tumor softening. The tumor mass occupied the majority of the left hepatic lobe, encased and pushed aside the right hepatic vein and displaced the portal vein and hepatic artery (Figure 1). The histopathology revealed an undifferentiated spindle cell sarcoma composed of relatively bland spindled cells arranged in short fascicles embedded in a myxoid stoma (Figure 2). There were regions of extensive tumor necrosis. Well-preserved tumor exhibited up to 9 mitoses per 50 high-power fields (Figure 3). Tumor cells were negative for pancytokeratin, smooth-muscle actin, desmin, S-100 protein, PGP 9.5, CD34, CD31, and c-kit. The combination of large tumor size, extensive tumor necrosis, and readily identifiable mitoses suggested this sarcoma was at least of intermediate grade. Bridging fibrosis and atrophy were present in the adjacent hepatic parenchyma. The common hepatic artery lymph node was negative for tumor. 

The patient did not receive any postoperative chemotherapy. The initial transplant was complicated by ductopenic rejection and the patient was retransplanted in October of 2002. The explant of the initial liver graft did not show any evidence of tumor recurrence. He has been on surveillance for tumor recurrence since then and has remained tumor-free for 10 years and 3 months since his last transplant. 

## 2. Discussion

Primary sarcomas of the liver are rare tumors which usually occur in the pediatric age group [1]. Angiosarcoma, leiomyosarcoma, and fibrosarcoma are the more common and better recognized liver sarcomas. Undifferentiated or embryonal cell sarcoma (UECS) is a relatively new clinicopathologic entity that was first described in 1978 by Stocker and Ishak [2]. These sarcomas are usually pediatric tumors occurring in the age group between 5 and 15 years and are very rare in adults. Only a handful of adult patients with UECS have been described in the literature. In a recent review on 67 adult patients with UECS, the median age of the study group was 25 with the oldest patient being 86 years old [1]. Another review reported a total of 51 adult cases of UECS in the past 50 years [3]. 

In most cases, symptoms related to the tumor are usually nonspecific with abdominal pain and abdominal mass reported to be the most common presenting complaint [3–5]. Generally, liver function tests and serum AFP were reported to be normal. In our patient, synthetic liver function was preserved and the noted portal hypertension was probably related to mechanical compression of the portal vein by the liver tumor.

On ultrasound, UECS usually appears as a hypoechoic solid mass [6]. CT characteristics include the presence of an enhanced peripheral rim, some solid portions at the periphery or adjacent to the septa, and a predominantly cystic or multicystic appearance [6, 7]. These features have at times led to this tumor being misdiagnosed as a benign cystic lesion like hydatid cyst [8]. Discrepancy between the solid appearance on sonography and cystic appearance on CT should raise suspicion for this tumor [6]. Most of the reported tumors have risen from the right lobe, but our patient had a left lobe tumor and it appeared predominantly solid on the CT scan.

UECS are usually large solitary tumors, measuring 10–20 cm in diameter, with an average weight of 1310 g [2]. The cut surface is grey-white, variegated and glistening, alternating with cystic gelatinous areas, and/or red and yellow areas of hemorrhage and necrosis. The tumor may be separated from the adjacent compressed parenchyma by a fibrous pseudocapsule. Microscopically, there are usually large areas of necrosis with areas of viable tumor, usually located near the edge of the mass. The viable tumor cells are stellate or spindle-shaped and has ill-defined outlines. They may be arranged either compactly or loosely in abundant myxoid matrix or fibrous stroma. The tumor cells often have irregular, hyperchromatic nuclei, numerous mitosis, and often bizarre giant cells. Multiple, varying-sized eosinophilic globules in the tumor cell cytoplasm are a characteristic feature. These globules are PAS-positive and diastase-resistant. Histological features usually show positivity for vimentin, *α*1-antichymotrypsin, *α*1-antitrypsin, and focal positivity for cytokeratin, desmin, *α*-SMA, muscle-specific actin, CD68, myoglobin, nonspecific enolase, S100, and CD34 [4, 9]. This suggests that though UECS is essentially an undifferentiated tumor there may be areas of partial differentiation [10]. The tumor we report here was actually negative for all the above stains.

The prognosis of UECS is usually dismal with early reports suggesting a median survival of less than 1 year [2]. More recent reports on children have suggested longer survival with aggressive multimodality therapy and complete surgical resection [11]. UECS is so rare in adults that prognosis and survival are difficult to determine and are based mainly from small case series. In a review of 67 adult patients with UECS, the median survival was 29 months and survival was longest in patients who underwent complete tumor resection and adjuvant chemotherapy [1]. On literature review, we found only one adult patient who achieved long-term recurrence-free survival of 12 years after undergoing complete resection [12]. None of the adult patients reported in, literature have actually undergone liver transplantation as the curative procedure. Liver transplantation for UECS has been described in two pediatric patients, but in both patients it was only used as a backup procedure after relapse from initial treatment [13, 14]. 

Liver transplantation has been used for a variety of uncommon hepatic tumors such as primary angiosarcoma of the liver. However, this is the first adult case report of an undifferentiated sarcoma of the liver to be treated successfully with a liver transplantation and achieving long-term survival. Since UECSs are usually large tumors and their resectability can be limited by local invasion, liver transplantation can be considered an alternative curative surgery in selected patients.
